# CyanoCyc cyanobacterial web portal

**DOI:** 10.3389/fmicb.2024.1340413

**Published:** 2024-01-31

**Authors:** Lisa R. Moore, Ron Caspi, Douglas A. Campbell, John R. Casey, Sophie Crevecoeur, David J. Lea-Smith, Bin Long, Naaman M. Omar, Suzanne M. Paley, Nicolas M. Schmelling, Alejandro Torrado, Jonathan P. Zehr, Peter D. Karp

**Affiliations:** ^1^SRI International, Menlo Park, CA, United States; ^2^Mount Allison University, Sackville, NB, Canada; ^3^Lawrence Livermore National Laboratory, Physical and Life Sciences Directorate, Livermore, CA, United States; ^4^Watershed Hydrology and Ecology Research Division, Environment and Climate Change Canada, Burlington, ON, Canada; ^5^School of Biological Sciences, University of East Anglia, Norwich, United Kingdom; ^6^Department of Plant Pathology and Microbiology, Texas A&M University, College Station, TX, United States; ^7^Krauts and Sprouts, Duesseldorf, Germany; ^8^Institute of Plant Biochemistry and Photosynthesis, University of Seville and Spanish National Research Council, Sevilla, Spain; ^9^Ocean Sciences Department, University of California, Santa Cruz, Santa Cruz, CA, United States

**Keywords:** CyanoCyc, cyanobacteria, genomes, metabolism, bioinformatics, database, curation, biotechnology

## Abstract

CyanoCyc is a web portal that integrates an exceptionally rich database collection of information about cyanobacterial genomes with an extensive suite of bioinformatics tools. It was developed to address the needs of the cyanobacterial research and biotechnology communities. The 277 annotated cyanobacterial genomes currently in CyanoCyc are supplemented with computational inferences including predicted metabolic pathways, operons, protein complexes, and orthologs; and with data imported from external databases, such as protein features and Gene Ontology (GO) terms imported from UniProt. Five of the genome databases have undergone manual curation with input from more than a dozen cyanobacteria experts to correct errors and integrate information from more than 1,765 published articles. CyanoCyc has bioinformatics tools that encompass genome, metabolic pathway and regulatory informatics; omics data analysis; and comparative analyses, including visualizations of multiple genomes aligned at orthologous genes, and comparisons of metabolic networks for multiple organisms. CyanoCyc is a high-quality, reliable knowledgebase that accelerates scientists’ work by enabling users to quickly find accurate information using its powerful set of search tools, to understand gene function through expert mini-reviews with citations, to acquire information quickly using its interactive visualization tools, and to inform better decision-making for fundamental and applied research.

## Introduction

Cyanobacteria are some of the oldest, most diverse and ecologically important microbial phototrophs on Earth. With the rapid development of genomic tools applied to cyanobacterial research and the growing interest in cyanobacteria as sources of marketable secondary metabolites and as carbon-neutral, bioengineering platform organisms, there is an increasing need for reliable knowledgebase databases. Unfortunately, the long-term maintenance of such databases has been a challenge. For instance, CyanoBase (Nakao et al., 2010; Fujisawa et al., 2017), the central knowledgebase for the cyanobacterial community, is frequently unavailable and has limited bioinformatics tools. In addition, it is well-known that funding for life-science databases often ceases after a limited time-period, thus undermining the long-term viability of these resources.

The lack of information regarding cyanobacterial genomics, as well as concerns over the instability of many disparate bioinformatics resources inspired a roundtable discussion at the 14th Workshop on Cyanobacteria, which took place at Michigan State University in June 2022, and an *ad hoc* committee was formed to explore alternatives. After months of discussion, the committee recommended development of a new cyanobacterial database collection integrated with bioinformatics tools to serve the community’s needs. CyanoCyc – a cyanobacterial database portal that resides within the larger, well-established BioCyc collection (Karp et al., 2019) – was developed, and the committee was re-defined to serve as an Advisory Board, providing invaluable input during CyanoCyc’s implementation.

The CyanoCyc web portal couples a rich collection of cyanobacterial genome databases with powerful and easy-to-use bioinformatics tools to enable studies of cyanobacteria from molecules to metabolic models, and from basic biology to synthetic biology. CyanoCyc contains 277 cyanobacterial genomes across 56 genera and covers a vast range of phenotypic types, from soils to freshwater and the marine environment. Five of the databases, *Synechocystis* sp. PCC 6803 substr. Kazusa, *Synechococcus elongatus* PCC 7942, *Synechococcus* sp. WH8102, *Prochlorococcus marinus* CCMP1375 (*P. marinus* SS120) and *Prochlorococcus marinus pastoris* CCMP1986 (*P. marinus* MED4), were further improved by curators, who manually corrected annotation errors in the genomes and entered information gathered from more than 1,765 publications (Table 1). The curated information ranges from experimentally elucidated gene functions to regulatory events and metabolic pathways. Overall, the CyanoCyc databases span a wide range of information, including gene functions; protein complexes; protein features; Gene Ontology annotations (Ashburner et al., 2000); metabolic pathways, reactions, and metabolites; gene essentiality datasets; and regulatory interactions.

**Table 1:** The five manually curated databases in CyanoCyc and some statistics on their content.

	*Synechocystis*	*Synechococcus elongatus* PCC 7942	*Synechococcus* sp. WH 8102	*P. marinus* SS120	*P. marinus* MED4
Genome size	3,956,956	2,750,104	2,434,428	1,751,080	1,657,990
Genes	3,679	2,772	2,578	1,931	1,840
Pathways	256	243	219	191	234
Publications	1,028	422	184	76	55
Summaries	384	216	234	115	154

Genes and Pathways: Counts of genes and pathways in each database. Publications are the number of publications cited in and unique to each database, from which data were curated. Summaries quantifies the curator-authored summary information, as textbook-page-equivalents, in each database. A report that includes additional points of comparison is available from the BioCyc Comparative Analysis tool. Data is from BioCyc version 27.1, released on August 28, 2023.

By integrating such a diverse range of information types, CyanoCyc satisfies a large set of information-seeking needs of cyanobacterial scientists. For example, given a gene name, CyanoCyc enables users to determine the function of its product, what complex, if any, the product participates in, and retrieve any curated regulatory influences on the gene and its product. If the gene encodes an enzyme, CyanoCyc identifies reactions the enzyme catalyzes and the pathway(s) in which these reactions participate. Orthologs of the gene in other organisms can be readily identified and compared.

CyanoCyc also contains a large number of bioinformatics tools that are integrated with all the genome databases and simplify access to information, produce visual outputs, and perform multiple computational analyses. As part of the BioCyc collection, users also have access to the more than 20,000 microbial databases, making the bioinformatics tools even more useful. Some of these tools (all CyanoCyc tools are also present in BioCyc), comprise:

- Searches that enable users to quickly find desired information, including:
  - Boolean searches, e.g., find all genes in a given region of the chromosome whose products lie in a given molecular-weight range and are found in the membranes
  - Very fast single-organism BLAST searches
  - Sequence pattern searches
- A genome browser that enables graphical exploration of the genome and comparisons across multiple genomes
- A metabolic network browser that enables exploration of pathways and metabolic capabilities
- A suite of omics-data analysis tools that enables quick and novel analyses of transcriptomics, proteomics, and metabolomics data
- Various tools for between-database comparisons
- SmartTables that allow analytical spreadsheet capabilities linked to information within BioCyc and the ability to bring in your own data, e.g., omics data, shared and exported information
- A feedback tool that enables users to suggest database corrections and updates

In this paper, we describe the methodology for creation of the new databases, the manual curation process by in-house curators and cyanobacteria community experts, the enhancements to information presentation and bioinformatics tools inspired by the Advisory Board, and the propagation of ortholog-based information to additional cyanobacterial genomes. We also present examples of what types of research can be performed with the bioinformatics tools available through CyanoCyc and BioCyc as a whole.

We address the sustainability of CyanoCyc by offering the website through a subscription model. Even with a sequenced genome in hand, our knowledge of gene function, metabolism, regulation, and other aspects of the cyanobacteria unfolds over a time period of decades. With the last update to CyanoBase occurring in 2017 (Fujisawa et al., 2017), and with the gradual decay in operational fitness of CyanoBase, the difficulty of maintaining and sustaining databases over long periods of time is evident. For many years the National Science Foundation (NSF) funded most database grants for only a single grant cycle. NSF did fund the TAIR (The Arabidopsis Information Resource) database for multiple grant cycles. Eventually NSF phased out TAIR’s funding, compelling them to shift to a subscription model, which has been successful for more than a decade (Check Hayden, 2013; Reiser et al., 2016). Like TAIR, BioCyc converted to a subscription model in 2016. Because BioCyc supports genome databases for a wide set of bacteria, including the cyanobacteria within the CyanoCyc portal, BioCyc is able to spread many of its costs, such as website operations, across a larger user community, thus keeping overall subscription prices relatively low. Given that no other high-quality, integrated database for the cyanobacteria has emerged and been maintained since the first cyanobacterium was sequenced 27 years ago (Kaneko et al., 1996), we hope the community will support the CyanoCyc subscription model for sustainability.

## Methods

This section describes the methods used to create and curate new CyanoCyc Pathway/Genome Databases (PGDBs). To construct each database, an annotated genome was imported from RefSeq (O'Leary et al., 2016) and enriched by importing additional information from other databases, such as protein features from UniProtKB (UniProt Consortium, 2023), and by running a series of computational inferences stored in the database to produce results, such as computational prediction of metabolic pathways from its sequenced genome. We collaborated with cyanobacteria research experts on the manual curation process and to make enhancements to BioCyc bioinformatics tools.

### Creating new CyanoCyc databases

In BioCyc parlance, a Tier 3 PGDB is one that is purely computationally generated; a Tier 2 database is manually curated after it is generated; and a Tier 1 database includes at least a 1-person-year of curation, and sometimes much more. For example, our EcoCyc database for *E. coli* K-12 has undergone several person-decades of curation based on information derived from more than 42,000 publications (Karp et al., 2023).

Creation of an uncurated (Tier 3) CyanoCyc database begins with computational ingestion of a RefSeq annotated genome followed by a series of computational inferences. Several components of SRI’s Pathway Tools software (Karp et al., 2021) were used to predict additional information available in the database:

- Predicted metabolic reactions and pathways were imported from the MetaCyc reference pathway database (Caspi et al., 2020)
- Operons were predicted using a combination of features including distance and functional relatedness between adjacent genes
- Protein complexes were predicted using keyword analysis of gene product names
- Transport reactions were predicted from keyword analysis of transporter names
- Pathway hole fillers (genes whose products are predicted to catalyze pathway steps that currently have no associated enzyme) were predicted
- Orthologs were computed between each protein in the organism and the proteomes of all other BioCyc organisms
- Pfam domain (Mistry et al., 2021) searches were run across the proteome; matching domains were saved as protein features

After these computational predictions were performed, we imported several types of data (when available) from UniProtKB into the new PGDBs:

- Protein features such as metal-ion binding sites and enzyme active sites
- Gene Ontology annotations
- UniProtKB accession numbers
- UniProt functional annotations not present in the RefSeq annotation

Additionally, links to databases such as UniProt, AlphaFold (Jumper et al., 2021), and NCBI Protein (Sayers et al., 2022) were created to provide access to additional information not included in BioCyc Tier 3 PGDBs.

### Manual curation and review process

The manual curation process is complex and involves error correction, entry of missing information, and resolution of conflicts in the literature. At first, computationally built databases may be error-laden. There are multiple reasons for the errors, including incorrect protein functional annotations introduced by RefSeq. For example, incorrect annotation may include the term ‘ubiquinone’ for a cyanobacterial quinone synthesis enzyme based on sequence similarity to a non-cyanobacterial enzyme – even though cyanobacteria do not produce ubiquinone. Incorrect inferences by the PTools software include attachment of the wrong reactions to gene products, annotation of generic reactions (classes of multiple reactions), or prediction of pathways that do not exist in the organism. During the process of manual curation, the curators perform many tasks, some of which are not dependent on the literature, and these include filling in missing gene names not comprised in the genome annotations, creating protein complexes not predicted by PTools; importing metabolic pathways from MetaCyc that are known to exist in the organism but were not computationally predicted due to missing or incorrect annotation; and correcting computationally predicted data such as transcription units based on conflicting experimental evidence. By far the most time-consuming part of manual curation is the addition of material obtained from the literature: adding new information about gene function, regulatory information, and novel reactions and pathways, and writing mini-reviews with citations.

All five Tier 2 curated species were subject to a careful manual curation process. Additionally, the most recently curated database for *Synechocystis* sp. PCC 6803 was also reviewed over a 2-month period by a panel of eight cyanobacterial researchers to ensure high-quality data. An invitation to participate in curating the *Synechocystis* sp. PCC 6803 genome database on CyanoCyc was emailed to over 600 cyanobacteria researchers who attended several cyanobacteria-related conferences and was posted to social media sites to provide opportunity for a broad range of participants. The curation review panel was selected from those who expressed strong interest and the ability to commit the time to the curation review process. The goals of the review panel were to review protein function and pathway information within the database, which the reviewers identified in a collaborative, online meeting, suggest specific changes and/or additional information using the “Provide Feedback” button, and log what information was reviewed in a spreadsheet so we could keep track of the process. The actual curation was performed by CyanoCyc curators. The researchers contributed expertise in fields such as biosynthetic pathways, electron transport reactions, pilus formation, and DNA methylation/restriction enzymes.

### Ortholog-based propagation of annotations

Manually curated gene and protein data from the five Tier 2 curated databases were propagated to the corresponding orthologs in Tier 3 (uncurated) databases for other cyanobacteria. This procedure (Paley et al., 2021) leverages limited curation resources across a wide set of databases, and thereby raises the quality of the entire collection. Several measures were employed to protect against potential errors. Firstly, data was only propagated from an organism to closely related organisms. Secondly, propagation was performed only if four criteria were met: the source protein had undergone some manual curation, as indicated by a non-computational evidence code or curation credit; a single candidate ortholog could be identified in the target strain; the two genes had sequence similarity value of *p* <1e-10; and gene lengths differed by no more than 10%.

For ortholog pairs meeting all the criteria for propagation, the target gene and protein names were updated to match the source, with all replaced values recorded as part of the gene’s history visible to users. All reaction and GO term assignments were propagated. In addition, if the source protein was a member of a complex, the corresponding complex was created in the target database. Finally, any newly predicted pathways based on the updated annotations were imported, and (after examination of the database transaction log) any manually deleted pathways predicted in both source and target organisms based on the same level of evidence were also deleted from the target database.

## Results

### Databases and curation process

The CyanoCyc portal contains 277 cyanobacterial genomes spanning 56 genera covering the extensive morphological and ecological diversity of cyanobacteria from the toxin-producing freshwater cyanobacterium *Microcystis aeruginosa* NIES-843 (Kaneko et al., 2007) to the soil cyanobacterium *Nostoc calcicola* FACHB-389 (Zhu et al., 2017), and to the fast-growing *Synechococcus elongatus* PCC 11801 (Jaiswal et al., 2018) of interest in genetic engineering applications. We added seven additional genomes that the Advisory Board members suggested were particularly useful to the research community, and we will add more genomes by user request via our request form (URL = https://cyanocyc.org/request-new-pgdb.shtml) for new genomes. So far, we have manually curated five databases, all of which describe picocyanobacteria, including the freshwater organisms *Synechococcus elongatus* PCC 7942 and *Synechocystis* sp. PCC 6803 substr. Kazusa, and the marine organisms *Synechococcu*s sp. WH8102, *P. marinus* SS120, and *P. marinus* MED4 (Table 1). Both *Synechococcus elongatus PCC 7942* and *Synechocystis* sp. PCC 6803 are exceptionally well-studied model organisms and are commonly used in metabolic engineering (Jaiswal et al., 2022). *Synechococcu*s sp. WH8102 is representative of organisms with a high phycourobilin chromophore composition that are highly abundant in oligotrophic oceanic waters (Scanlan et al., 2009). *P. marinus* is the dominant photosynthetic organism in most tropical and temperate open ocean ecosystems. The high-light clade I ecotype, *P. marinus* MED4, is characteristic of the surface layer, and the low-light clade II/III ecotype, *P. marinus* SS120, is typical of organisms adapted to lower light conditions (Biller et al., 2015).

The curation review process of the *Synechocystis* sp. *PCC 6803* database was carried out by eight cyanobacteria experts who focused on many metabolic pathways, including the Entner-Doudoroff and hydrocarbon biosynthetic pathways, such as alkane biosynthesis; proteins and reactions, such as the photoactive orange carotenoid protein; the photosynthetic electron transport reactions, such as the pathway of electron transport in the thylakoid membrane and in the plasma membrane; various transport processes, such as the linear electron flow through plastocyanin; and the pilin proteins, such as those found in the type IV pilus. The experts suggested new text describing information in peer-reviewed publications about proteins, reactions, and pathways that were not yet curated in the database, and also recommended a few corrections. For some topics, the reviewers reached out to other colleagues to obtain additional information. Experts are credited for their specific reviews below the summaries on the information pages.

In the future, we aim to curate additional cyanobacteria that are deemed important to the fundamental understanding of cyanobacterial biology, ecology, and potential applications in biotechnology. Potential candidates include: *Nostoc* sp. PCC 7120, a model nitrogen-fixing, heterocyst-containing strain (Zeng and Zhang, 2022); *Microcystis aeruginosa*, which forms harmful algal blooms (Harke et al., 2016); *Arthrospira platensis*, commercially cultivated for its nutritional value as the food product *Spirulina* (Gentscheva et al., 2023); and *Gloeocapsa biformis*, which causes streaks and stains on monuments and buildings (Macedo et al., 2009).

### Curated databases

The CyanoCyc Advisory Board members suggested we create publicly accessible SmartTables (Table 2) listing CyanoCyc organisms based on their phenotypic subgroups (e.g., filamentous organisms, heterocyst formers, etc.). In addition, we created SmartTables including the five curated cyanobacteria in CyanoCyc (Cyanobacteria List: Curated Cyanobacterial Databases) and a representative list of the phylogenetically diverse cyanobacteria that are frequent subjects of publication (Cyanobacteria List: Representative Cyanobacteria) to enable comparisons across a wide set of organisms. When performing Sequence Pattern Searches or CyanoCyc comparative operations, these SmartTables can be used to define the set of organisms. Thus, for example, one could run a metabolic network comparison across the list of organisms stored in the SmartTable “Cyanobacteria list: Nitrogen Fixing organisms” using the Comparative Analysis tool and choosing the Pathways table (Tools > Analysis > Comparative Analysis). We also extended the SmartTables facility so it is possible to use an “Add Property Column” menu to include columns for phenotypic properties such as the aerobicity of the organism and the date the organism was collected.

**Table 2:** SmartTables listing CyanoCyc organisms grouped according to their phenotype.

SmartTable name	Number of databases
Cyanobacteria List: Filamentous Organisms	127
Cyanobacteria List: Heterocyst Formers	66
Cyanobacteria List: Nitrogen Fixing Organisms	89
Cyanobacteria List: Obligate Symbionts	9
Cyanobacteria List: Picocyanobacteria	60
Cyanobacteria List: Unicellular Cyanobacteria	140

These SmartTables, created February 27, 2023, can be found in the listing of all Public SmartTables. Since the title of each table starts with “Cyanobacteria List,” it is easy to search for (“Filter”) them. Each Public SmartTable indicates the criteria used to include or exclude specific cyanobacteria databases and provides time stamps for when they are updated.

### Ortholog-based propagation

We propagated orthologs from the two *Prochlorococcus* databases to 16 other *Prochlorococcus* strains; the *Synechocystis* database to six other *Synechocystis* sp. PCC 6803 and closely related strains, the *Synechococcus elongatus* PCC 7942 to four other *Synechococcus elongatus* and closely related strains; and the *Synechococcus* sp. WH8102 to four marine *Synechococcus* strains (Table 3). The propagated genes include a number of cases in which functions were inferred for genes of previously unknown function. For example, in the uncurated database for *Synechocystis* sp. PCC 6714, gene RS07570 was previously annotated simply as “hypothetical protein.” After propagation from its ortholog in *Synechocystis* sp. PCC 6803 Kazusa, gene RS08070, the previously hypothetical protein acquired the name *vte3*, the function tocopherol cyclase, and two associated reactions, which filled previous holes in the vitamin E biosynthesis pathway in *Synechocystis* sp. PCC 6714. The corresponding gene page (URL = https://biocyc.org/gene?orgid=GCF_000478825&id=D082_RS07570) includes an ortholog link and shows the summary description from the ortholog in the source database, as well as its history of updates.

**Table 3:** The number of Tier 3 databases to which the orthologs were propagated from the curated database and the average numbers of curated genes propagated to orthologs in the recipient databases.

Curated database	Tier 3 databases receiving ortholog information	Average number of propagated genes per database (range)
*Synechocystis* sp. PCC 6803	6	313 (288–323)
*Synechococcus elongatus* PCC 7942	4	486 (441–529)
*Synechococcus* sp. WH8102	4	410 (120–526)
*P. marinus* SS120	16	53 (46–64)
*P. marinus* MED4	16	18 (16–19)

Links to the curated database are provided. Data for *Synechocystis* sp. PCC 6803 is from BioCyc version 27.5, released on December 8, 2023; data for other databases is from BioCyc version 27.1, released on August 28, 2023.

### Community-driven database enhancements

At the suggestion of the CyanoCyc Advisory Board members, we made multiple enhancements to CyanoCyc. We created the CyanoCyc.org home page and an Introduction to CyanoCyc page that contains information about the databases and tools and provides links to other cyanobacterial resources. We added the carboxysome to the cellular compartment ontology used in the databases to more accurately depict carboxysome-associated reactions and pathways. To accommodate the ongoing changes to cyanobacterial taxonomic nomenclature and enable greater ease in finding cyanobacterial species based on older (and often more commonly used) names, the Advisory Board identified such names for many cyanobacterial species. These older names are listed on each organism’s Summary Statistics (“home”) page and can be used when selecting a database (e.g., “*Nostoc* var*iabilis* ATCC 29413” was entered as a synonym for *Trichormus variabilis* ATCC 29413). Improvements to some bioinformatics tools suggested by the Advisory Board members have also been incorporated, e.g., we have extended the Sequence Pattern Search capabilities of the website so that searches can be performed not just against the genome and proteome of a single organism but against a set of peptides listed in a SmartTable, or against the full proteomes of multiple organisms listed in a SmartTable such as those in Table 2.

We will continue to develop the CyanoCyc portal to address the needs of the cyanobacterial community. For example, we could import data on transcription start sites, terminators, and transcription factor binding sites, if available; add connections to other databases, such as CyanoSource that has a library of barcoded knockout mutants for *Synechocystis* sp. PCC 6803 mutants; or generate a Flux Balance Analysis metabolic model for *Synechocystis* sp. PCC 6803 that users can run. These future improvements to the databases will be explored with input from the Advisory Board and the larger community.

Cyanobacterial scientists can contribute to CyanoCyc in several ways. At the bottom of every CyanoCyc web page is a link “Report Errors or Provide Feedback” which can be used to report data errors or errors in the software tools, and to submit suggestions for improved functionality. For those interested in making a longer term series of enhancements to the databases, please contact us through the above link or via email to the corresponding author. We will provide you with access to our web-based database editing tools that curators use to make updates to the databases.

### What can you do with CyanoCyc?

Here we explore some of the operations the CyanoCyc bioinformatics tools provide, grouped into five categories: genomic, metabolic, regulatory, omics-data analytic, and comparative operations, and provide guidance about how to access the tools and generate the example figures.

**Figure 1:**
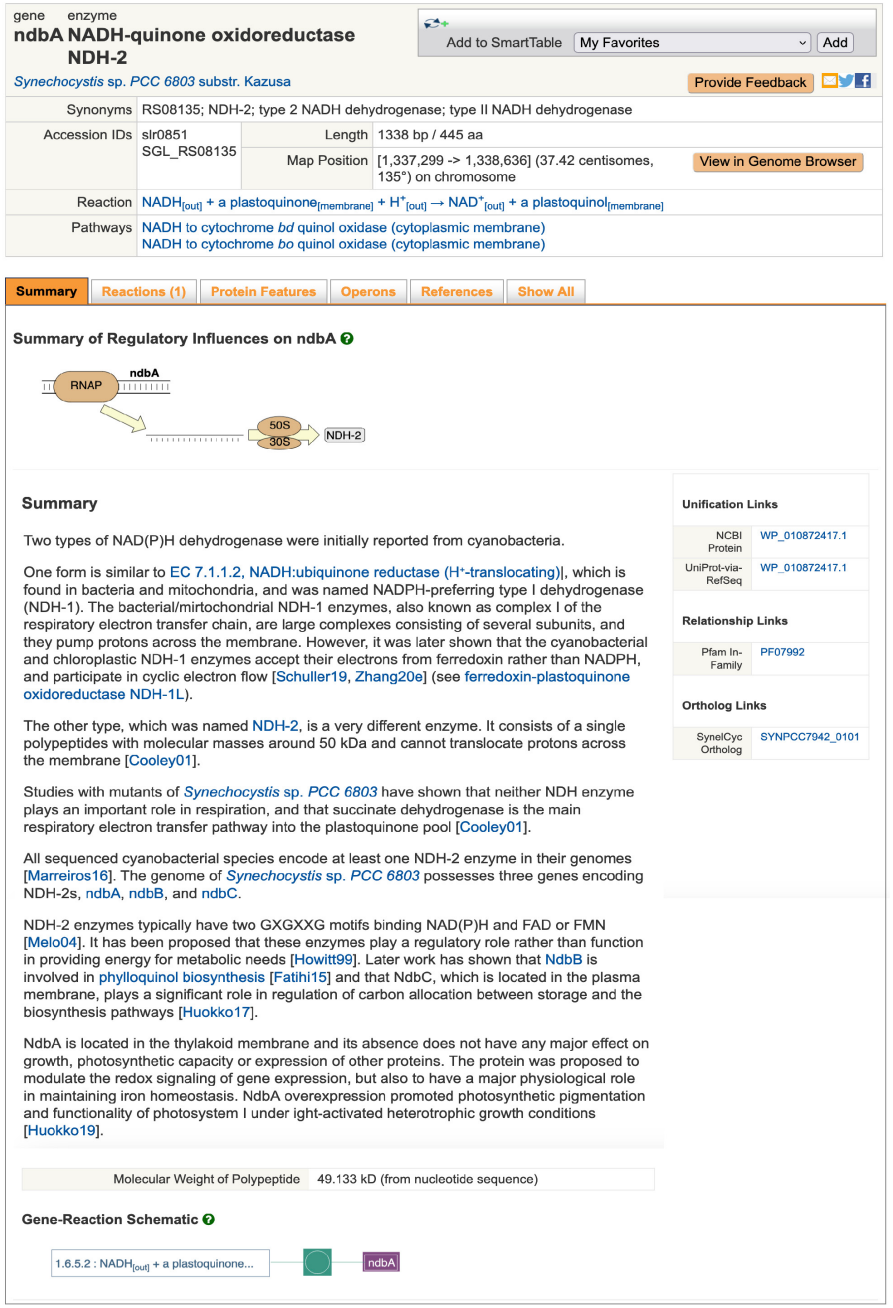
Example of page describing a gene, *ndbA* of *Synechocystis* sp. PCC 6803, encoding a subunit of a type II NADH Dehydrogenase. URL: https://cyanocyc.org/gene?orgid=GCF_000009725&id=SGL_RS08135. To generate this page online: (1) Open CyanoCyc.org in a web browser. (2) Click button “Change Current Database,” then type “kazusa” into the box under “Select a Database,” and click on “Synechocystis sp. PCC 6803 substr. Kazusa” that appears just beneath, then click OK. (3) Just below and to the right of the “Change Current Database” is the text entry box for searching – type “ndbA” into that box and press Enter to reach the page for that gene.

Genomic operations include searching for genes and their products by name using the Search in Current Database field or (via the advanced search Tools > Search > Search Genes, Proteins, or RNAs) by additional properties including pI and protein features, such as amino acid metal binding sites. The results are shown as Gene/Protein Information pages (for instance, ndbA, Figure 1). From the gene page, the user can retrieve the nucleotide sequence of the coding region, or of a user-defined genome region, or the amino-acid sequence of the gene product. The site supports BLAST searches against all of BioCyc and against individual genomes. The latter are quite rapid, approximately 15 s. Sequence pattern searches for nucleotide and amino-acid sequences are also supported based on the PatMatch sequence-pattern language (Yan et al., 2005). CyanoCyc provides both linear and circular genome browsers that enable the user to zoom from the sequence level to a single-page view of an entire replicon. The user can also execute sequence alignments among multiple selected sequences. A poster-size genome map diagram can be generated using Tools > Genome > Generate Genome Poster.

**Figure 2:**
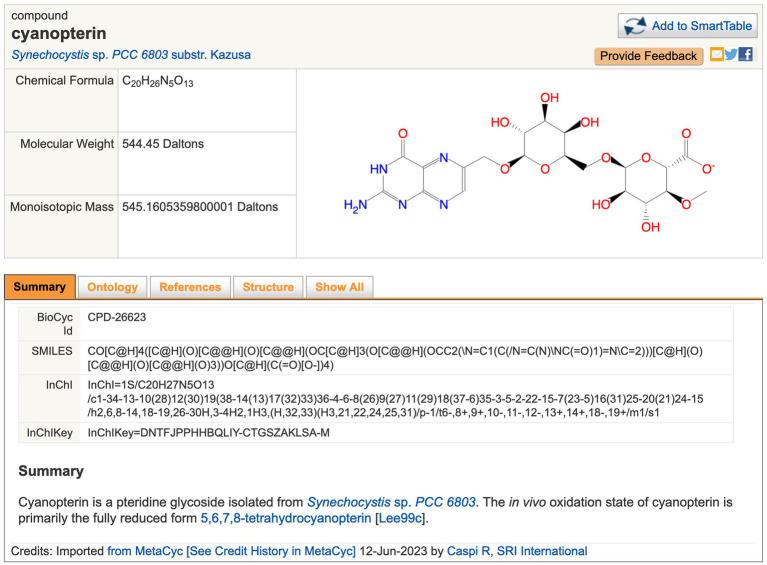
Example of page describing the metabolite cyanopterin. URL: https://cyanocyc.org/compound?orgid=GCF_000009725&id=CPD-26623. To get to this page: (1) Follow the instructions provided for Figure 1, except enter “cyanopterin” into the Search in Current Database field and press Enter. (2) Several search results are shown; click “cyanopterin” under the Compounds section to go to the metabolite page shown here.

**Figure 3:**
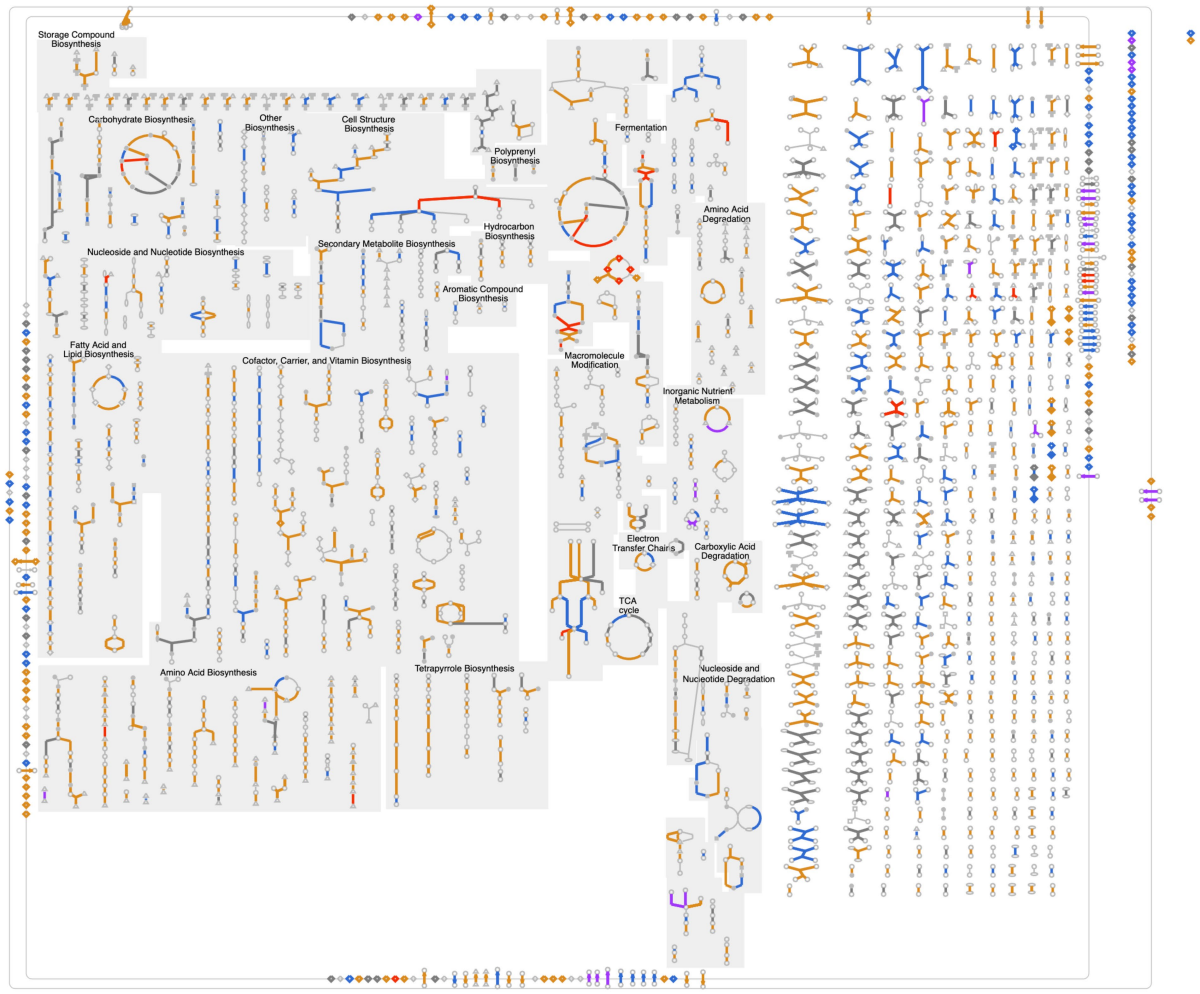
A cellular overview for the cyanobacterium *Synechocystis* sp. PCC 6803 (URL: https://biocyc.org/overviewsWeb/celOv.shtml?orgid=GCF_000009725) with superimposed omics data from an experiment studying the effect of excess iron on the organism (one time point from GEO dataset # GDS3741, Houot et al., 2007). To generate this figure: (1) Within the *Synechocystis* sp. PCC 6803 substr. Kazusa PGDB, go to the Tools drop down menu, then select on Cellular Overview underneath the Metabolism section. (2) Once on the Cellular Overview page, click on Import Data from GEO under the OPERATIONS panel to the right of the page. (3) In the pop-up window that opens, enter the GEO dataset # in the Keywords box and press Go to connect to the dataset. (4) Highlight the data to display, such as GSM84561, and press Submit. (5) Change the Colors Scheme, *Y*-Axis scale and run the Animation using the Omics Control Panel that pops up once the data is overlaid. A flow diagram of these instructions is also available in Supplementary Figure S1; these can be used as a starting off point for displaying other omics data on the Cellular Overview.

Metabolic operations include searches for metabolites, reactions, and metabolic pathways using names, or by other criteria including metabolite monoisotopic mass, chemical formula, and InChI string using advanced search tools such as Tools > Search Compounds. The results are shown as information pages for metabolites (e.g., cyanopterin, Figure 2), reactions (e.g., EC 4.1.2.13), and pathways (e.g., echinenone and zeaxanthin biosynthesis and linear electron flow with plastocyanin). Metabolic pathway diagrams are automatically drawn by the software with user selection of the detail level that can drill down from key reactant names to regulators to metabolite structures. Three tools are provided for exploring complete metabolic networks (see Tools > Metabolism). (1) The Cellular Overview provides zoomable, searchable, organism-specific metabolic network diagrams for every CyanoCyc organism (Figure 3), and a printable poster-size metabolic chart diagram can be generated from this diagram using Tools > Metabolism > Generate Metabolic Map Poster. (2) The Metabolic Route Search tool enables the user to specify starting and ending metabolites of interest; the tool generates alternative minimal-cost routes through the network that connects these metabolites. (3) The Metabolic Network Explorer tool is similar to Metabolic Route Search but supports interactive, incremental exploration: the user starts at a metabolite of interest and incrementally adds one reaction at a time, building up a user-guided pathway from one metabolite to another.

Regulatory operations consist of visualization tools for various regulatory relationships. Gene pages contain a regulation summary diagram that depicts all the available regulatory influences on the gene and its product, including transcriptional, translational, and post-translational regulation. Gene pages for transcription factors contain a Regulon tab that depicts all operons controlled by the transcription factor, provided that information has been curated. A regulatory network browser tool (see Tools > Genome) depicts the full regulatory network of organisms containing significant numbers of regulatory interactions. The number of regulatory interactions curated in the CyanoCyc databases is, as yet, relatively small.

Omics analytic operations are available for transcriptomic, proteomic, and metabolomic data.

**Figure 4:**
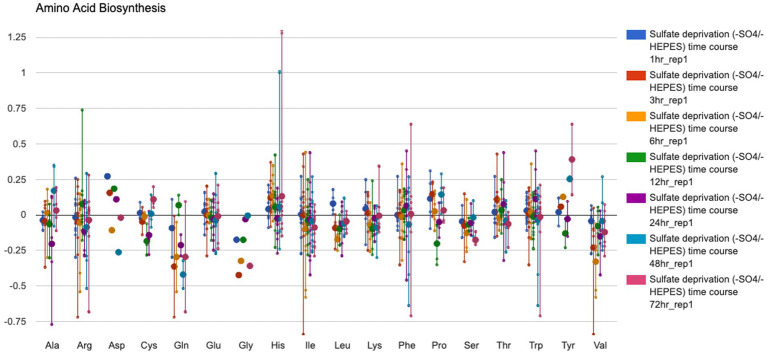
The Omics Dashboard data analysis tool showing transcription levels of *Synechocystis* sp. PCC 6803 genes involved in amino acid biosynthesis from a culture grown with a low amount of sulfur (GEO dataset # GDS3745, Zhang et al., 2008). To generate this figure: (1) Follow instructions for Figure 3 except use the GEO dataset # GDS3745 and highlight rep 1 for time points 1–72 h under sulfate deprivation; press Submit. (2) Select “Export to Dashboard” at the bottom of the Omics Control Panel to open the data in a new browser window. (3) In the Biosynthesis data panel, mouse over the data area for AA Syn and click to open a new window showing the average gene expression data for the enzymes involved in biosynthesis of each amino acid.

The tools include:

- Enrichment analysis for metabolic pathways for transcriptomic, proteomics and metabolomic data
- Visualization of transcriptomic, proteomic, and metabolomic data on individual pathways, on multi-pathway diagrams called pathway collages, and on a full metabolic network diagram for the organism. The latter visualization can be animated for datasets containing multiple observations.
- Visualization of transcriptomic, proteomic, and metabolomic data on a system-oriented diagram called the Omics Dashboard (Figure 4), which depicts the activation levels of every subsystem within the cell and enables the user to probe the expression levels of individual subsystems in more detail.

**Figure 5:**
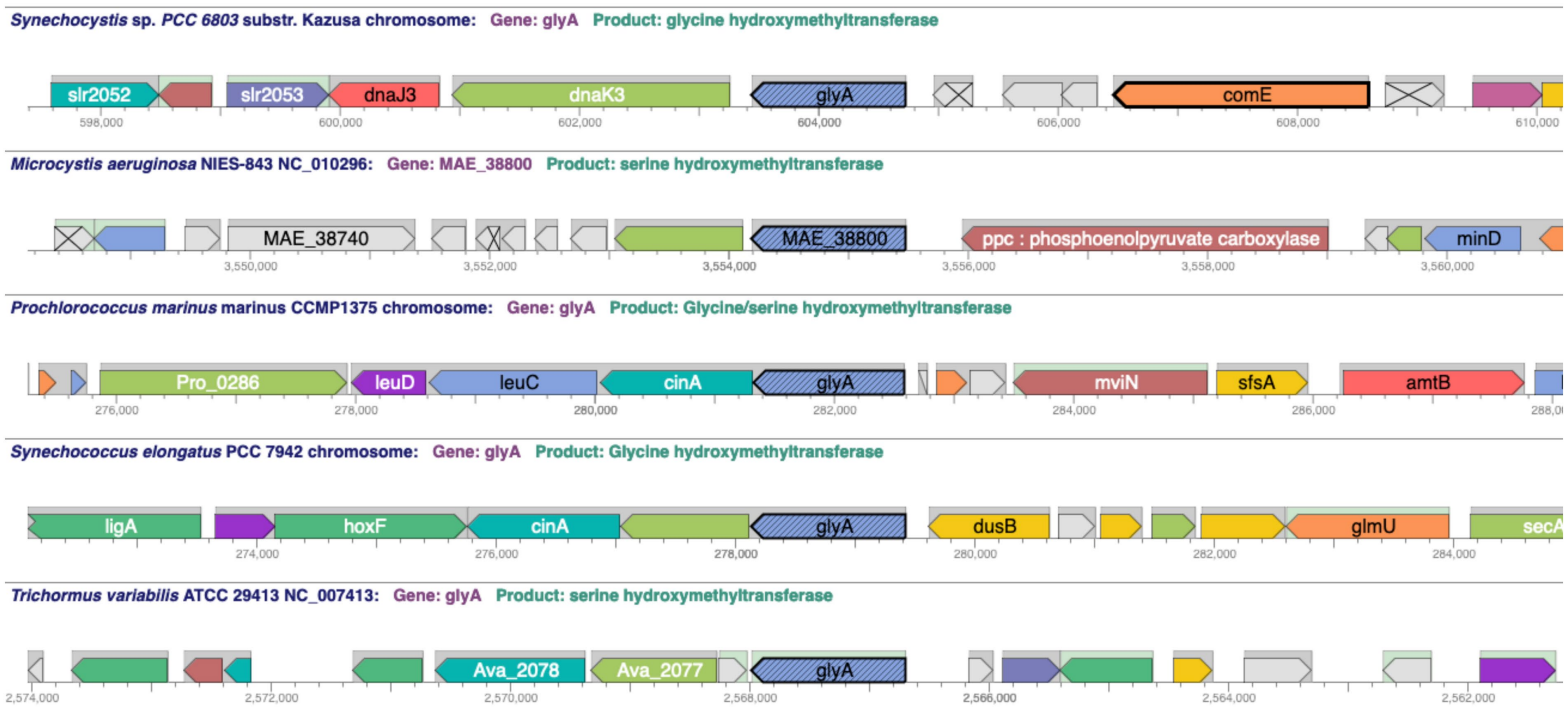
A comparative genome browser showing *glyA* gene orthologs across five cyanobacterial species and the genome context around the *glyA* orthologs using the “Align in Multi-Genome Browser” tool. URL: https://cyanocyc.org/genbro/ortho.shtml?lead-orgid=GCF_000009725&lead-genes=SGL_RS04690&orgids=GCF_000010625,SS120,SYNEL,GCF_000009725,GCF_000204075. To make this figure: (1) With the *Synechocystis* sp. PCC 6803 substr. Kazusa PGDB selected as the current database, enter the string “*glyA*” into the “Search in Current Database” field and press Enter. (2) Choose “Align in Multi-Genome Browser” under the Operations panel on the right-hand side of the gene/protein information page. (3) A window opens allowing the user to “Specify List of Organism Databases” to be used in the alignment. (4) On the left-hand side, enter the name of organism desired, e.g., *Microcystis aeruginosa* NIES-843, in the field, highlight (if necessary), then click “Add →” to put it into the Current Selection list on the right-hand side. (5). When your list is complete, push the “OK” button at the bottom right to generate the figure.

Comparative operations include several tools that leverage the ortholog information in CyanoCyc. The genome browser has a comparative mode that enables the user to align replicons from multiple organisms at an orthologous gene group to compare the genome context around those genes (Figure 5). This has been used to determine the extent of conservation in genome structure for cytochrome c6 genes (Torrado et al., 2023) and *crtE* genes (Satta et al., 2022) in diverse cyanobacteria. A related tool produces a table listing of a group of orthologous genes including the gene name, annotated function, and a diagram of the containing operon.

A suite of comparative analysis tools (Tools > Analysis > Comparative Analysis) generates tables comparing statistics across a selected set of genomes, ranging from comparisons of proteomes (e.g., identifying proteins that have no orthologs in the other organisms), pathways, reactions, metabolites, and transporters.

**Figure 6:**
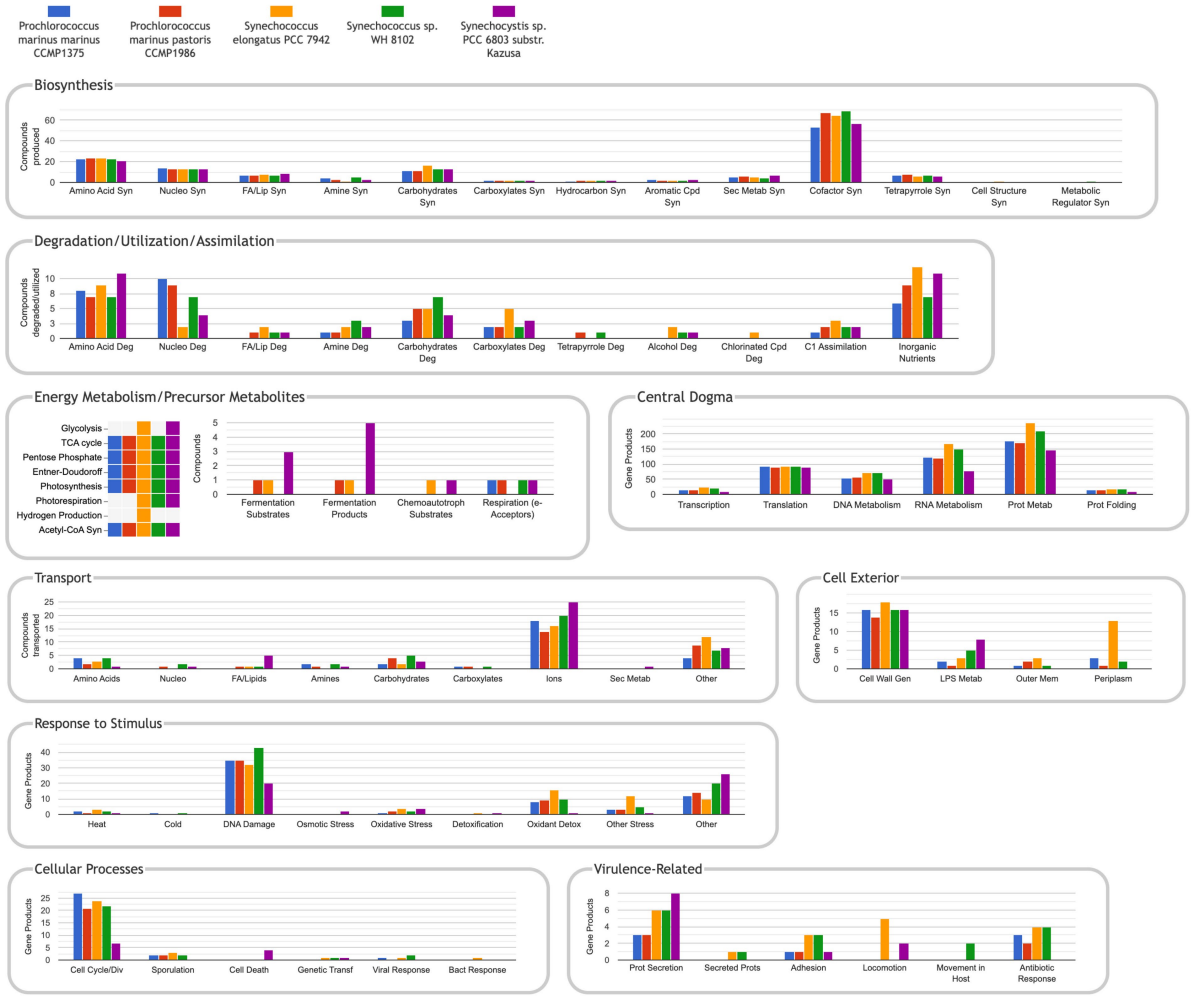
The comparative genome dashboard for the five manually curated databases listed in Table 1. URL: https://cyanocyc.org/dashboard/genome-dashboard-compare.shtml?orgids=(SS120+MED4+SYNEL+SYNWH8102+GCF_000009725). To get to this page: (1) Within the *Synechocystis* sp. PCC 6803 substr. Kazusa PGDB, go to the Tools drop down menu, then select “Comparative Genome Dashboard” under the “Analysis” section. (2) Click on “Select Organisms for Comparison to open the “Specify List of Organism Databases” popup. (3) Enter the names of the organisms desired and “Add” to the “Current Selection,” then press “OK.”

The Comparative Genome Dashboard is a unique graphical tool that enables visual comparisons of the metabolic capabilities of a set of organisms including their biosynthesis, catabolism, and transport capacities (Figure 6). If the user wishes to see a more detailed view within a given subsystem (e.g., amino acid biosynthesis), they can drill down to produce an elaborate listing of which metabolites within that subsystem each organism can synthesize.

### How to learn more

The Introduction to CyanoCyc link on the CyanoCyc portal provides additional information. Users new to BioCyc and its bioinformatics tools should watch the BioCyc 2-Minute Tutorials and then refer to the longer tutorials on that same web page and to the Website User’s Guide.

## Summary

The CyanoCyc portal to cyanobacterial genomes within the larger BioCyc organismal collection of genome databases was developed at the request of and with the input from cyanobacterial researchers to provide a reliable, integrative, rich database for research purposes. The CyanoCyc Advisory Board made essential suggestions for improving various aspects of the cyanobacterial information and bioinformatics tools. An expert review panel of cyanobacterial researchers contributed valuable curation suggestions for the newly curated Tier 2 *Synechocystis* sp. PCC 6803. This collaborative CyanoCyc effort between the cyanobacteria research community and the BioCyc curators and developers has provided an essential resource for diverse cyanobacteria research and biotechnological fields. It also provides a path for future collaborations of BioCyc and other organismal research scientists to increase resources for genomic and metabolic information and bioinformatic resources.

## Data availability statement

The original contributions presented in the study are included in the article/Supplementary material, further inquiries can be directed to the corresponding authors.

## Author contributions

LM: Conceptualization, Data curation, Methodology, Project administration, Writing – review & editing, Formal analysis, Visualization, Investigation, Supervision. RC: Data curation, Methodology, Visualization, Writing – review & editing, Investigation. DC: Data curation, Writing – review & editing. JC: Data curation, Writing – review & editing, Conceptualization. SC: Writing – review & editing. DL-S: Data curation, Writing – review & editing. BL: Conceptualization, Data curation, Writing – review & editing. NO: Data curation, Writing – review & editing. SP: Investigation, Methodology, Software, Visualization, Writing – review & editing. NS: Data curation, Writing – review & editing. AT: Conceptualization, Writing – review & editing. JZ: Conceptualization, Writing – review & editing. PK: Conceptualization, Data curation, Formal analysis, Investigation, Methodology, Project administration, Resources, Software, Supervision, Writing – original draft, Writing – review & editing.
