# Supplementary material for: CyanoCyc cyanobacterial web portal
Source: Front Microbiol. 2024 Jan 31;15:1340413. doi: 10.3389/fmicb.2024.1340413 (PMC10864581; doi:10.3389/fmicb.2024.1340413)
Supplement: Supplementary file 1 [file Presentation_1.PPTX]

## Slide 1
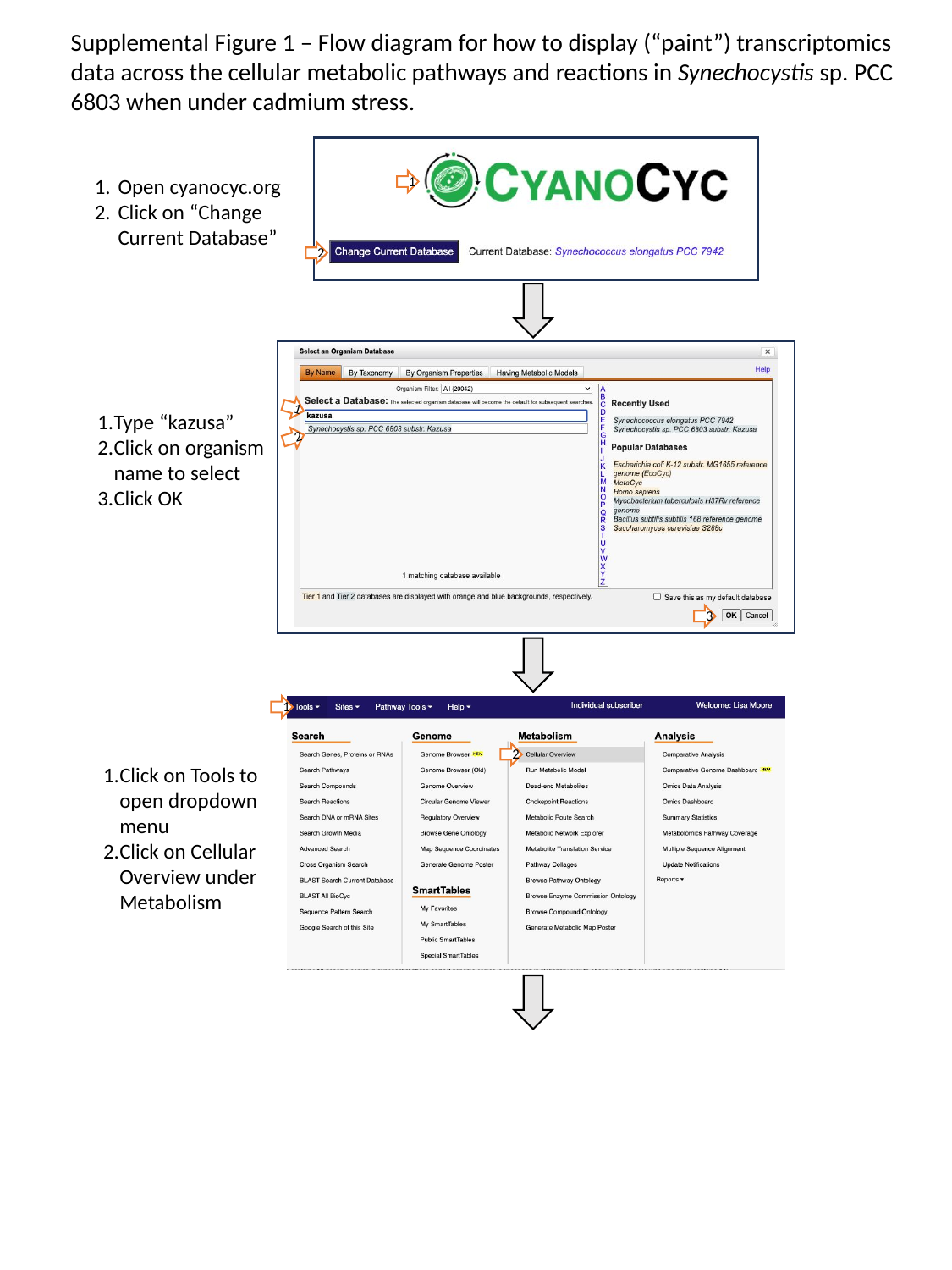

Supplemental Figure 1 – Flow diagram for how to display (“paint”) transcriptomics data across the cellular metabolic pathways and reactions in Synechocystis sp. PCC 6803 when under cadmium stress.
1
2
Open cyanocyc.org
Click on “Change Current Database”
1
2
3
Type “kazusa”
Click on organism name to select
Click OK
1
2
Click on Tools to open dropdown menu
Click on Cellular Overview under Metabolism

## Slide 2
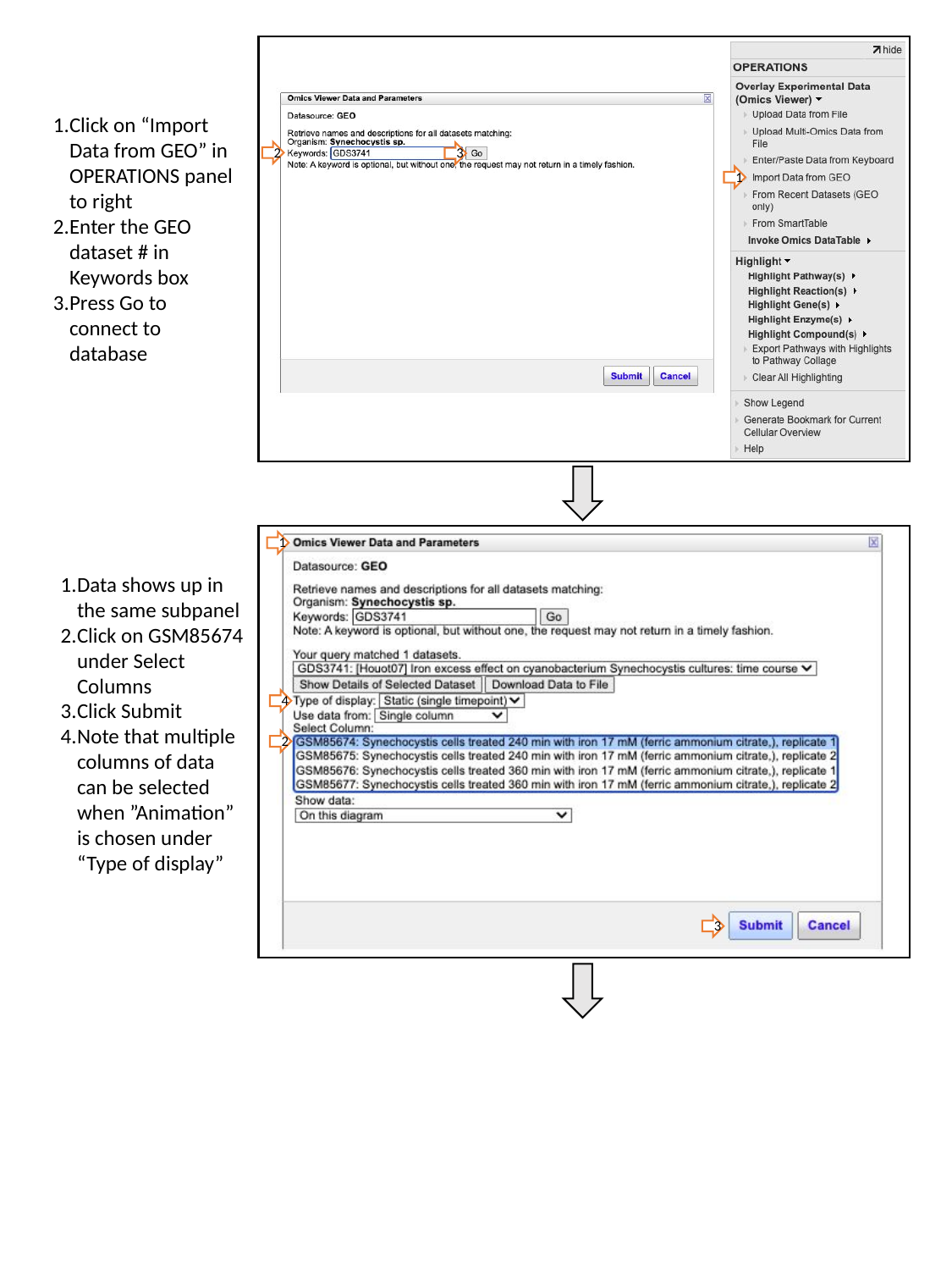

2
3
1
Click on “Import Data from GEO” in OPERATIONS panel to right
Enter the GEO dataset # in Keywords box
Press Go to connect to database
1
4
2
3
Data shows up in the same subpanel
Click on GSM85674 under Select Columns
Click Submit
Note that multiple columns of data can be selected when ”Animation” is chosen under “Type of display”

## Slide 3
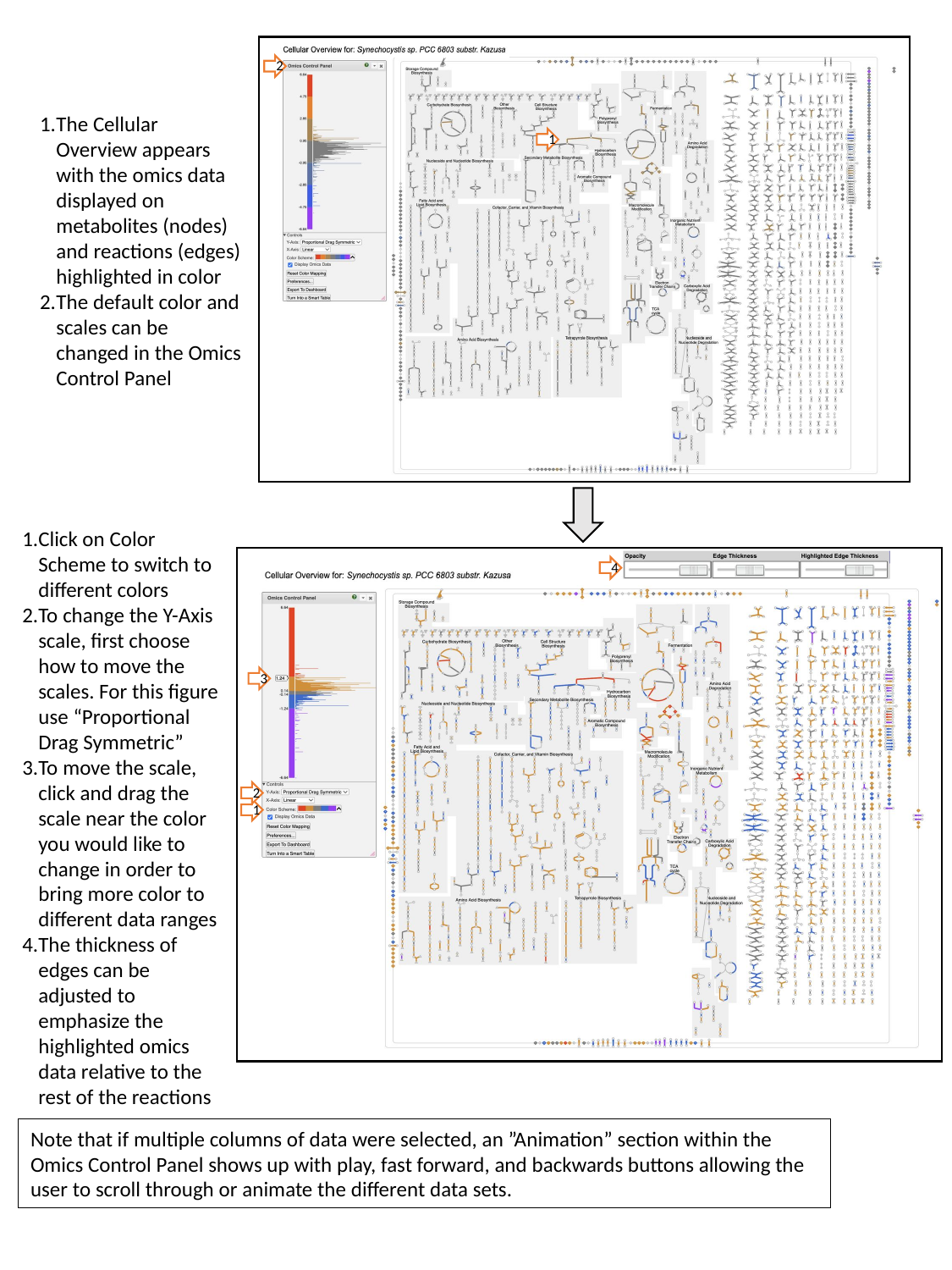

2
The Cellular Overview appears with the omics data displayed on metabolites (nodes) and reactions (edges) highlighted in color
The default color and scales can be changed in the Omics Control Panel
1
Click on Color Scheme to switch to different colors
To change the Y-Axis scale, first choose how to move the scales. For this figure use “Proportional Drag Symmetric”
To move the scale, click and drag the scale near the color you would like to change in order to bring more color to different data ranges
The thickness of edges can be adjusted to emphasize the highlighted omics data relative to the rest of the reactions
4
3
2
1
Note that if multiple columns of data were selected, an ”Animation” section within the Omics Control Panel shows up with play, fast forward, and backwards buttons allowing the user to scroll through or animate the different data sets.
